# Leisure-time physical activity in Amazonian pregnant women and offspring birth weight: A prospective cohort study

**DOI:** 10.1371/journal.pone.0265164

**Published:** 2022-03-16

**Authors:** Maíra B. Malta, Paulo A. R. Neves, Bárbara H. Lourenço, Maria Helena D. A. Benício, Guilherme L. Werneck, Marcia C. Castro, Marly A. Cardoso, for the MINA-Brazil Study Working Group

**Affiliations:** 1 Department of Nutrition, School of Public Health, University of São Paulo, São Paulo, Brazil; 2 Postgraduate Program in Epidemiology, Federal University of Pelotas, Pelotas, Brazil; 3 Institute of Studies in Public Health, Federal University of Rio de Janeiro, Ilha do Fundão–Cidade Universitária, Rio de Janeiro, RJ, Brazil; 4 Department of Epidemiology, Social Medicine Institute, State University of Rio de Janeiro, Maracanã, Rio de Janeiro, RJ, Brazil; 5 Department of Global Health and Population, Harvard T.H. Chan School of Public Health, Boston, MA, United States of America; Universiti Malaya, MALAYSIA

## Abstract

Compelling evidence supports the current international recommendation of at least 150 min/week of leisure-time physical activity (LTPA) during pregnancy. However, the potential relationship between LTPA and birth weight (BW) remains unclear in low- and middle-income countries. The purpose of this study was to examine the association between LTPA during pregnancy and offspring BW in an Amazonian population. Prospective cohort study was carried-out with 500 pregnant women and their offspring followed-up in the MINA-Brazil study, which was conducted in Cruzeiro do Sul, Western Brazilian Amazon. LTPA was assessed in the second (mean 19.6, SD 2.4 weeks) and third (mean 27.8, SD 1.6 weeks) gestational trimesters by a standardised interview and categorised according to the recommended cut-off of at least 150 min/week. We calculated offspring BW z-scores by sex and gestational age. We then explored the effect of LTPA during pregnancy on offspring BW, the association between LTPA and small-for-gestational-age (SGA) or large-for-gestational-age (LGA) births, and the mediating role of excessive and insufficient gestational weight gain (GWG). At least 150 min/week of LTPA during the third gestational trimester was associated with an offspring BW decrease of -0.35 z-score (95% CI: -0.65, -0.05) or -147.9 grams (95% CI: -260.9, -14.8), without increasing the frequency of SGA foetuses either in the second or third gestational trimester (p > 0.05). Excessive GWG mediated the effect of LTPA on the offspring BW (indirect effect = -0.05 z-score [95% CI: -0.10, -0.00] or -34.7 grams (95% CI: -66.1, -3.3]). This mediation effect was not observed for insufficient GWG. LTPA in the third, but not in the second, trimester of pregnancy was inversely associated with offspring BW without increasing the frequency of SGA, an effect that was partly mediated by excessive GWG.

## Introduction

Substantial scientific evidence supports the current international recommendation of at least 150 min/week of leisure-time physical activity (LTPA) during pregnancy [1, 2]. However, few pregnant women perform physical activity [3, 4]. Population-based studies in the United Kingdom and the United States estimated only 3% to 15% of women are physically active during pregnancy [5–7]. Cohort studies showed that almost 50% of women performed regular exercise before pregnancy, but this figure declined at the second to third gestational trimester [7–9]. A Brazilian study that evaluated time changes in LTPA from 2004 to 2015 found that a small proportion of participants achieved the recommended level, and that LTPA remained stable for the first and second trimesters before decreasing in the last trimester [10].

The benefits of LTPA during pregnancy on maternal and child health include reduced risk of excessive gestational weight gain (GWG) [11, 12], gestational diabetes mellitus [11, 13], gestational hypertensive disorders (mainly preeclampsia) [11, 14], preterm birth [11, 15], foetal macrosomia, large-for-gestational age (LGA) newborns [16], and offspring obesity in adulthood [17]. A meta-analysis of randomised controlled trials (n = 4,909) found no evidence for an effect of LTPA on average birth weight (BW); however, high heterogeneity was detected across the trials. The same meta-analysis evaluated six cohort studies (n = 62,127 women) and showed LTPA had a small decreased effect on mean BW, with no heterogeneity [8, 11]. A recent meta-analysis comprising 72,694 participants (mainly white women) from a consortium of eight studies (seven European and one American) showed that every additional hour per week of moderate to vigorous physical activity in late pregnancy decreased BW by 6.4 grams with decreased risks of macrosomia and LGA by 4% and 3%, respectively; no association was found between LTPA in early pregnancy and BW [16].

The previous studies on LTPA and BW have been published in high income countries and included mostly white women. Evidence from other populations is necessary, especially given the current context of a dual burden of under and overnutrition and its association with social inequalities in low- and middle-income regions [18]. Therefore, this study aimed to examine the association between LTPA during pregnancy and offspring BW in Amazonian women.

## Materials and methods

### Study population

This study is based on data from the Maternal and Child Health and Nutrition in Acre (MINA-Brazil) prospective birth cohort, performed in the urban area of Cruzeiro do Sul in Western Brazilian Amazon, as described elsewhere [19]. The municipality is the second largest city in Acre State and had an estimated population of 87,600 inhabitants in 2018 [20], of which roughly 50% were women.

From February 2015 to January 2016, women with singleton pregnancies at 20 gestational weeks or less who were living in the urban area of the municipality [21] were screened by nurses in 13 primary health care units. The MINA-Brazil Study was approved by the Ethics Committee of School of Public Health, University of São Paulo (Protocol number 872.613, Nov 13^th^, 2014). The consent to participate was given through signature of the written informed consent. In case of teenage pregnants consent was obtained from the caregiver. For cases of illiteracy, a thumb finger print was obtained.

### Data collection

All eligible women were invited to participate in the MINA-Brazil study through phone calls or home visits by the research team. Upon acceptance and written informed consent, socio-economic and health data were collected by trained interviewers when the participants were 20 gestational weeks or less. During the interviews, the following data were gathered: maternal age, self-reported pre-pregnancy weight, maternal schooling, self-reported maternal skin colour, head of the family, living with a partner, maternal occupation, receipt of *Bolsa Família* conditional cash transfer programme, number of rooms in the household, and ownership of household assets. A household wealth index was constructed through principal component analysis, further used as a proxy of household income [22].

Following the interview, clinical evaluations were scheduled to collect anthropometric data and lifestyle behavioural information during pregnancy (smoking, frequency of food groups consumption, and physical activity). An ultrasound assessment was performed to estimate the gestational age, as described previously [23, 24]. These clinical evaluations were held in the second (mean gestational age of 19.6, SD 2.4 weeks, ranging from 7.7 to 23.9 weeks of pregnancy based on last menstrual period) and third trimesters of pregnancy (mean 27.8, SD 1.6 weeks, ranging from 24.1 to 35 weeks).

Using a questionnaire adapted from previous studies [25, 26], the frequency of food groups consumption in the past month was recorded with the following response options: never, 1 to 3 times per month, 1 to 3 times per week, 4 to 6 times per week, 1 time per day, 2 to 3 times per day, and ≥ 4 times per day. For the present analysis, we focused on the frequency of consumption of (i) fruits and vegetables and (ii) ultra-processed foods (ready-to-eat products, packaged attractively and marketed intensively, that require little or no preparation [27, 28], including soft drink/ sugared drinks and industrialised food as snacks, cookies, sweets) in the second and third trimesters of pregnancy, considering the following categories: no/monthly, weekly, or daily consumption.

All anthropometric measurements followed WHO recommendations [29]. We used a portable scale (Tanita Corporation, Tokyo, Japan), model UM061, with capacity for 150 kg and variation of 0.1 kg to measure the participants’ body weight. To measure height, we used a portable stadiometer (Alturaexata^®^, Belo Horizonte, Brazil) with an extension of 213 cm and a precision of 0.1 mm. Each anthropometric measurement was taken in duplicate, and the mean values were calculated.

Pre-gestational body mass index (BMI) was calculated using self-reported pre-pregnancy body weight and height measured in early pregnancy and classified according to the WHO criteria [29] for adult participants: underweight (< 18.5 kg/m^2^), normal weight (≥ 18.5 to < 25.0 kg/m^2^), overweight (≥ 25.0 to < 30.0 kg/m^2^), and obese (≥ 30 kg/m^2^). We used the Anthro Plus software [30] to classify the pre-pregnancy BMI z-scores of adolescent participants, which were in turn categorised as: underweight (z-score ≤ -2); normal weight (z-score > -2 to z-score <+1); overweight (z-score ≥ +1 to z-score <+2) and obese (z-score ≥ +2).

At birth, we gathered data from hospital records on prenatal care, anthropometric measures, and birth and offspring assistance: primigravida, number of antenatal care appointments, occurrence of gestational urinary tract infection, gestational diabetes, type of delivery, gestational age at birth, pre-birth maternal weight (in kg), offspring sex, and offspring BW (in kg). Offspring BW was obtained from the hospital records and was measured to the nearest 0.005 kg using a Toledo® Junior portable scale (São Bernardo do Campo, Brazil). All maternity ward staff involved in infant care received training on BW measurement, and the accuracy of the scales was checked daily by the research team of this study [21]. All data collection was performed by trained researchers using tablets programmed with CSPro software (https://www.census.gov/programs-surveys/international-programs.html) for data entry.

### Main exposures

LTPA was assessed using a questionnaire that was previously validated among Brazilian pregnant women [31]. LTPA was chosen as it is the domain most amenable to intervention and therefore more relevant for public health recommendations and based on some studies showing that even mild physical activity during pregnancy has an independent protective effect on low birth weight, preterm birth, and intrauterine growth restriction [32, 33]. To calculate time spent in minutes of LTPA per week, participants were asked during the clinical evaluations about type of LTPA (walking, dancing, running, cycling, swimming, playing football, and others), frequency (one to seven days per week), and duration (minutes in each practice) in a typical week. Subsequently, a dummy variable was created based on international recommendations of LTPA in pregnancy, considering whether ≥150 minutes of LTPA was achieved or not per week in each evaluation [34, 35]. Therefore, our primary exposure was based on whether the participant achieved 150 minutes per week of LTPA (i) in the second and (ii) in the third trimesters of pregnancy (no or yes), and (iii) at any time of the pregnancy (no or yes). Additionally, we explored whether the participant achieved moderate to intense 150 minutes per week of LTPA and as a continuous variable, in hours per week.

### Outcomes and potential mediator variables

We categorised offspring BW as follows: low BW < 2500 grams, normal BW ≥2500 to < 4000 grams, and foetal macrosomia ≥ 4000 grams. Z-scores and percentiles of offspring BW were calculated according to the international standards established by the Intergrowth-21^st^ Project in relation to the final gestational age, by sex [36] (https://intergrowth21.tghn.org/intergrowth-21st-applications/). Our primary outcome was the offspring BW (in z-scores and grams, as continuous variables). We also explored the percentiles of BW categorised in small-for-gestational-age (SGA) (percentile < 10) and LGA (percentile > 90). We calculated the total GWG (in kg) by subtracting the pre-birth gestational weight from the pre-pregnancy weight. The GWG was classified as insufficient (below the recommendation), adequate (according to recommendation), or excessive (above the recommendation) according to the Institute of Medicine guidelines based on the pre-pregnancy BMI: underweight (2.5–18 kg), normal weight (11.5–16 kg), overweight (7–11.5 kg) and obese (5–9 kg) [37]. Our mediator variables were excessive and insufficient GWG (no or yes).

### Statistical analysis

In descriptive analyses, we estimated the frequencies, means, and standard deviation of the baseline participant characteristics. The characteristics of participants included in the present analysis and of those lost to follow-up were compared using Student’s t-test and the chi-square test for continuous and categorical variables, respectively. The Shapiro-Wilk test was used to check the assumption of normality of continuous variables. We performed linear regression models to evaluate the effect of LTPA for at least 150 min per week during pregnancy on offspring BW z-score and in grams and to evaluate the effect of LTPA in hours per week on BW in grams. Multiple adjusted models were fitted with initial selection of covariates associated with the outcome at P < 0.20 in crude analyses, using hierarchical conceptual determinants of offspring BW [38]. At each level of determination, covariates associated with the outcome at P < 0.10 or conceptually relevant were retained in the final multiple adjusted models. Missing data were included in the multiple models by creating missing-value categories.

Additionally, we performed stratified analysis by maternal age (adolescent or adult) and classification of GWG (adequate, insufficient, and excessive). We also explored the association between the exposure and the frequency of SGA and LGA using chi-squared tests and logistic regression.

Mediation analyses were performed to investigate the extent to which any associations between maternal LTPA and BW (in z-score and grams) were mediated via GWG (excessive or insufficient). We fitted two regression models: logistic regression for the mediator and linear regression for BW. In order to obtain estimates of total effect (TE), controlled direct effect (CDE), and natural indirect effect (NIE), the models were fitted using Stata’s paramed command [39]. TE is the total effect of LTPA in pregnancy on BW, including the effect that occurs due to the increased risk of inadequate GWG; CDE is the effect of LTPA in pregnancy on BW, without any effect that occurs via the effect of LTPA on risk of inadequate GWG; and NIE is the effect of GWG on offspring BW, independent of any effect of LTPA. We used Stata 13.0 for data analysis (StataCorp, College Station, Texas, USA).

## Results

**Fig 1:**
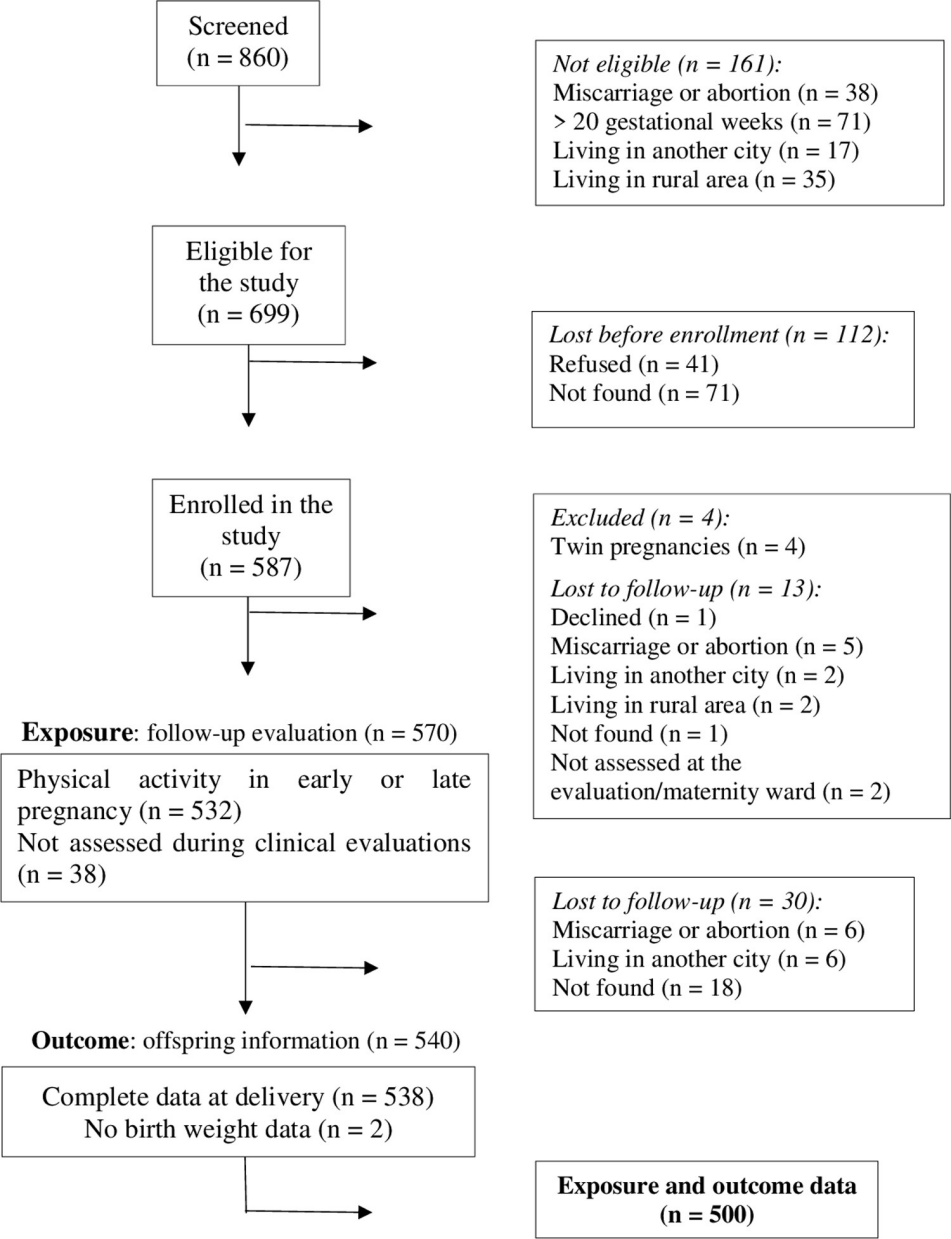
Flowchart of the recruitment and follow-up of the MINA-Brazil cohort.

Fig 1 presents the flowchart of recruitment and follow-up of participants in the MINA-Brazil study. Of 860 pregnant women initially screened, 699 were considered eligible. Of these, 41 pregnant women refused to participate in the study and 71 could not be located with the available contact information. Therefore, 587 pregnant women were enrolled in the cohort. A total of 570 pregnant women completed a clinical evaluation at the second or third trimesters of pregnancy. At birth, we collected data for 540 mother-infant pairs; of these, 500 women had data on physical activity during pregnancy (14.8% lost to follow-up). We did not observe significant differences in age (*p* = 0.728) or skin colour (*p* = 0.571) among the pregnant women who were successfully assessed during follow-up; however, the pregnant women lost to follow-up were poorer (*p* = 0.035) and had fewer years of schooling (*p* = 0.007) than those who were followed up.

Participant characteristics are presented in Table 1. Of the 500 women with available LTPA and BW data, 21% were adolescent, 78% self-reported their skin colour as brown, approximately 30% had less than ten years of schooling, about 42% achieved the recommendation of 150 minutes per week of LTPA before pregnancy, 36.86% reached the recommendation of moderate to intense intensity, and 30% and 40% had insufficient and excessive GWG, respectively.

**Table 1:** Characteristics of pregnant women at baseline and factors associated with the offspring birth weight z-score for gestational age in the MINA-Brazil birth cohort study.

Independent variables (n = 500)	N (%)	Offspring birth weight (z-score for gestational age)		
		β	(95% CI)	P
Wealth index	500 (100)	0.08	-0.03; 0.19	0.148
Number of rooms in the household	500 (100)	0.04	-0.00; 0.09	0.070
Living with a partner				
No	121 (22.40)	Reference		
Yes	388 (77.60)	0.20	-0.00 0.40	0.050
Head of the family				
Pregnant woman	65 (13.00)	Reference		0.283
Maternal schooling				
≤ 9 years	151 (30.2)	-0.11	-0.29; 0.08	0.253
> 9 years	349 (69.8)	Reference		
Maternal age	500 (100)	0.02	0.01; 0.03	0.010
Maternal skin colour				
White	69 (13.8)	Reference		
Brown	392 (78.4)	-0.02	-0.27; 0.22	0.845
Other (mainly black)	39 (7.8)	-0.18	-0.55; 0.20	0.360
Maternal occupation				
Paid job	218 (43.6)	Reference		0.656
*Bolsa Família* cash transfer programme				
No	300 (60.0)	Reference		
Yes	200 (40.0)	-0.16	-0.33; 0.02	0.076
Gestational diabetes (n = 488)				
No	482 (98.77)	Reference		
Yes	06 (1.23)	0.49	-0.28; 1.26	0.214
Primigravida				
No	274 (55.80)	Reference		
Yes	226 (45.20)	-0.23	-0.40; -0.06	0.008
Pre-pregnancy BMI^a^				0.011
Normal weight	305 (61.00)	Reference		
Underweight	37 (7.40)	-0.32	-0.64; 0.01	0.054
Overweight	121 (24.20)	0.17	-0.03; 0.37	0.104
Obesity	37 (7.40)	0.43	0.11; 0.75	0.010
Pre-pregnancy BMI in kg—mean (SD)	23.43 (4.32)	0.04	0.02; 0.06	<0.001
Gestational weight gain^b^ (n = 462)				0.002
Adequate	136 (29.44)	Reference		
Insufficient	138 (29.87)	-0.16	-0.38; 0.07	0.177
Excessive	188 (40.69)	0.30	0.09; 0.51	0.005
Fruit and vegetable consumption in the 2^nd^ trimester of pregnancy^c^ (n = 455)				0.001
No/Monthly	105 (23.08)	Reference		
Weekly	186 (40.88)	0.57	0.34; 0.80	<0.001
Daily	164 (36.04)	0.48	0.24; 0.71	<0.001
Fruit and vegetable consumption in the 3^rd^ trimester of pregnancy^c^ (n = 452)				0.621
No/Monthly	116 (25.66)	Reference		
Weekly	194 (42.92)	0.18	-0.04; 0.40	0.115
Daily	142 (31.42)	0.07	-0.17; 0.31	0.558
Ultra-processed food consumption in the 2^nd^ trimester of pregnancy^c^ (n = 455)				0.156
No/Monthly	211 (46.37)	Reference		
Weekly	130 (28.57)	-0.02	-0.23; 0.20	0.870
Daily	114 (25.05)	-0.17	-0.39; 0.05	0.132
Ultra-processed food consumption in the 3^rd^ trimester of pregnancy^c^ (n = 452)				0.040
No/Monthly	219 (48.45)	Reference		
Weekly	119 (26.33)	-0.25	-0.46; 0.03	0.024
Daily	114 (25.22)	-0.20	-0.42; 0.02	0.071
Smoking during pregnancy				
No	479 (95.80)	Reference		
Yes	21 (4.20)	-0.55	-0.96; -0.13	0.010
LTPA before pregnancy^d^				
150 minutes per week	210 (42,77)	Reference		
Less than 150 minutes per week	281 (57,23)	0.04	-0.13; 0.21	0.640

Totals may differ due to missing values.

^a^BMI: Body mass index, classified according to references proposed by WHO for adults (WHO, 1995) and reference curves for adolescents (DE ONIS et al., 2007).

^b^According to the Institute of Medicine guidelines, 2013.

^c^ 2^nd^ trimester of pregnancy: mean 19.6 (SD 2.4) weeks of pregnancy; 3^rd^ trimester of pregnancy: mean 27.8 (SD 1.6) weeks of pregnancy.

^d^Laisure-time physical activity tree months before pregnancy.

Regarding the exposure of interest, practice of physical activity during pregnancy, 80% of pregnant women were inactive (not getting any physical activity beyond basic movement from daily life) during the second and 71.7% in the third trimesters of pregnancy. Only 4.2% reached the recommendation of moderate to intense physical activity for 150 min/week in the second and 6.6% in the third trimesters of pregnancy. Considering the time spent for physical activities, without evaluating the intensity of this activity, we had only 7.3% that reached 150 min/week in the second and 9.5% in the third trimesters of pregnancy. At any time during pregnancy, 13.3% of the participants achieved the recommended time, and only 8.8% reached the recommendation including intensity. Around 2.4% of pregnant women were highly active (with more than 300 minutes of moderate to intense intensity physical activity per week) in the second or third trimester of pregnancy (S1 Table).

Among the offspring, 48% were female, 46% were born through caesarean section, 8% were preterm, and the mean gestational age at birth was 39.4 (Standard Deviation, SD = 1.7) weeks. The frequency of LGA and SGA were 11% and 9%, respectively. The mean offspring BW was 3,252.3 grams (SD 514.2, ranging from 1,410.0 to 5,060.0 grams) and the z-score was 0.04 (SD 0.96, ranging from -2.7 to 3.0 z-score).

Table 2 presents the effect of LTPA during pregnancy on offspring BW. Achieving 150 min/week of LTPA in the second or third trimester of pregnancy, as well as specifically in the third trimester of pregnancy, was negatively associated with BW in grams and according to z-score in adjusted models. Offspring BW was reduced by 137.9 grams (95% CI: -260.9, -14.8) and 124.8 grams (95% CI: -224.8, -25.6) in the third and when either second or third trimesters of pregnancy were considered, respectively. We did not find a significant association between LTPA and BW in the second gestational trimester. Despite the small sample size, simmilar results were found when considering the effect of 150 min/week of moderate to intense LTPA on offspring birth weight (S2 Table).

**Table 2:** Effect of leisure-time physical-activity (LTPA) during pregnancy on offspring birth weight in the MINA-Brazil cohort.

		**Offspring birth weight (in z-score for gestational age)** ^b^	
		Crude Model	Adjusted Model^c^
	N (%)	β (95% CI)	β (95% CI)
LTPA in 2^nd^ trimester of pregnancy^a^			
150 minutes per week	33 (7.3)	-0.33 (-0.67; 0.02)	-0.28 (-0.62; 0.05)
Less than 150 minutes per week	422 (92.7)	Reference	Reference
LTPA in 3^rd^ trimester of pregnancy^a^			
150 minutes per week	43 (9.5)	**-0.35 (-0.65; -0.05)**	**-0.35 (-0.65; -0.05)**
Less than 150 minutes per week	409 (90.5)	Reference	Reference
LTPA in 2^nd^ or 3^rd^ trimester of pregnancy^a^			
150 minutes per week	66 (13.2)	**-0.35 (-0.60; -0.10)**	**-0.33 (-0.56; -0.09)**
Less than 150 minutes per week	434 (86.8)	Reference	Reference
		**Offspring birth weight (in grams)** ^**d**^	
		Crude Model	Adjusted Model^c^
	N (%)	β (95% CI)	β (95% CI)
LTPA in 2^nd^ trimester of pregnancy^a^			
150 minutes per week	33 (7.3)	-93.4 (-280.4; 93.6)	-104.5 (-243.2; 34.2)
Less than 150 minutes per week	422 (92.7)	Reference	Reference
LTPA in 3^rd^ trimester of pregnancy^a^			
150 minutes per week	43 (9.5)	**-233.0 (-394.3; -71.3)**	**-137.9 (-260.9; -14.8)**
Less than 150 minutes per week	409 (90.5)	Reference	Reference
LTPA in 2^nd^ or 3^rd^ trimester of pregnancy^a^			
150 minutes per week	66 (13.2)	**-186.2 (-318.8; -53.5)**	**-124.8 (-224.1; -25.6)**
Less than 150 minutes per week	434 (86.8)	Reference	Reference

^a^ 2^nd^ trimester of pregnancy: mean 19.6 (SD 2.4) weeks; 3^rd^ trimester of pregnancy: mean 27.8 (SD 1.6) weeks.

^b^Z-scores of birth weight for gestational age calculated according to the Intergrowth-21st Project standard [36].

^c^Adjusted model: adjusted by determinants in distal level (number of rooms in the household, household wealth index, living with a partner); intermediate level (pre-pregnancy body mass index, age, primigravida), and proximal level (frequency of fruit and vegetable consumption and ultra-processed food consumption, smoking during pregnancy).

^d^Controlled for newborn sex and gestational age at birth.

LTPA as a continuous variable (in hours per week) was not associated with BW in grams in the adjusted model, either in the second trimester of pregnancy (β: -4.62 hours/week, 95% CI: -28.29, 19.05) or the third trimester of pregnancy (β: -14.95 hours/week, 95% CI: -36.83, 6.93). The effect of LTPA during pregnancy on BW z-scores stratified by maternal age groups (adult and adolescents) and GWG were also examined. The observed association was stronger among adult participants, regardless of second or third trimester of pregnancy (β: -0.44 z-score, 95% CI: -0.71, -0.17). However, there was no association between LTPA and BW in adolescent participants. Considering the classification of GWG, achieving the LTPA recommendation was inversely associated with BW z-score among infants born to women who had excessive GWG (β: -0.48 z-score, 95% CI: -0.95, -0.01). LTPA was not associated with BW z-score when women had adequate or insufficient GWG (S3 Table).

**Fig 2:**
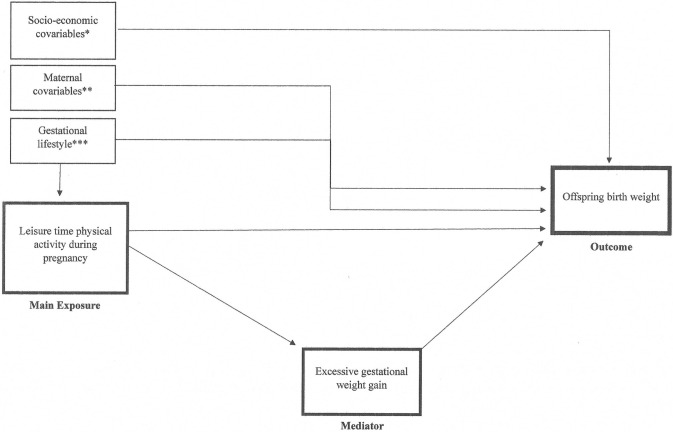
Proposed causal diagram among leisure time physical activity (LTPA) during pregnancy, gestational weight gain (GWG) and offspring birth weight. *Socioeconomic status baseline confounders included number of rooms in the household, household wealth index, living with a partner. ** Maternal covariables: pre-pregnancy BMI, age, primigravida. *** Gestational lifestyle: frequency of fruit and vegetable consumption and ultra-processed food consumption, smoking during pregnancy.

Table 3 presents the results of the estimates of direct and total effects of the association between LTPA and offspring BW as mediated through excessive and insufficient GWG. In models with excessive GWG as a mediator (Fig 2), while the TE of achieving 150 min per week of LTPA reduced on average the BW by 187.1 grams (*p* = 0.029), the CDE was equivalent to a reduction of 152.4 grams (*p* = 0.028). Similar findings were evident when the associations were mediated by insufficient GWG: with a TE of an average reduction in grams of 187.0 (*p =* 0.024), and a CDE of reduced by 180.7 grams (*p =* 0.009). We observed an additional mediated effect due to excessive GWG, with a NIE of -34.7 in grams (*p* = 0.030). No evidence of mediation through insufficient GWG was observed, considering a NIE of -6.4 grams (*p* = 0.659).

**Table 3:** Estimates of direct effect and effect mediated through excessive and insufficient gestational weight gain of the association between leisure-time physical activity (LTPA) and offspring birth weight in the MINA-Brazil cohort study.

	**Offspring birth weight (z-score for gestational age)** ^**b**^		
	Controlled direct effect β (95% CI)	Natural indirect effect β (95% CI)	Total effect β (95% CI)
**Excessive gestational weight gain**			
LTPA in 2^nd^ or 3^rd^ trimester of pregnancy^a^			
150 minutes per week	**-0.28 (-0.53, -0.03)**	**-0.05 (-0.10, -0.00)**	**-0.33 (-0.63, -0.04)**
Less than 150 minutes per week	Reference	Reference	Reference
**Insufficient gestational weight gain**			
LTPA in 2^nd^ or 3^rd^ trimester of pregnancy^a^			
150 minutes per week	**-0.33 (-0.58, -0.07)**	-0.01 (-0.05, 0.03)	**-0.33 (-0.62, -0.05)**
Less than 150 minutes per week	Reference	Reference	Reference
	**Offspring birth weight (in grams)**		
	Controlled direct effect β (95% CI)	Natural indirect effect β (95% CI)	Total effect β (95% CI)
**Excessive gestational weight gain**			
LTPA in 2^nd^ or 3^rd^ trimester of pregnancy^a^			
150 minutes per week	**-152.4 (-288.6, -16.2)**	**-34.7 (-66.1, -3.3)**	**-187.1 (-354.9, -19.3)**
Less than 150 minutes per week	Reference	Reference	Reference
**Insufficient gestational weight gain**			
LTPA in 2^nd^ or 3^rd^ trimester of pregnancy^a^			
150 minutes per week	**-180.7 (-316.8, -44.5)**	-6.4 (-34.7, 22.0)	**-187.0 (-349.7, -24.4)**
Less than 150 minutes per week	Reference	Reference	Reference

^a^ 2^nd^ trimester of pregnancy: mean 19.6 (SD 2.4) weeks; 3^rd^ trimester of pregnancy: mean 27.8 (SD 1.6) weeks.

^b^Z-scores of birth weight for gestational age calculated according to the Intergrowth-21st Project standard [36].

^c^Adjusted model: adjusted by determinants in distal level (number of rooms in the household, household wealth index, living with a partner); intermediate level (pre-pregnancy body mass index, age, primigravida), and proximal level (frequency of fruit and vegetable consumption and ultra-processed food consumption, smoking during pregnancy).

## Discussion

This prospective cohort study among Amazonian women showed that LTPA in the third trimester of pregnancy, but not in the second trimester of pregnancy, was inversely associated with offspring BW. Achieving at least 150 min/week of LTPA in the third trimester of pregnancy was associated with decreased offspring BW without increasing the frequency of small-for-gestational age foetuses. This association was significantly mediated by excessive GWG, but not by insufficient GWG.

To the best of our knowledge, this is the first prospective study from a middle income country to examine the effect of LTPA on offspring BW in pregnant women and investigate the mediation effect of GWG.

This investigation was performed in a challenging area of the Brazilian Amazon using methods routinely assessed for quality to ensure data reliability. The prospective study design minimised risk of recall bias. Considering the high prevalence of excessive (40%) and insufficient (30%) GWG in our study population, we could separately investigate possible mediation by each form of inadequate GWG. This is also the first study using BW z-scores according to the international standards of the Intergrowth-21^st^ Project [36] to assess the association between LTPA and BW.

Despite these strengths, there are some limitations of this study. First, the data on physical activity was self-reported and did not consider intensity due to the small sample of women who reached the recommendation of 150 min/week of moderate to intense physical activity (4.2% in the second trimester and 6.6% in the third trimester of pregnancy). The finding of no correlation between LTPA as a continuous measure (in hours per week) and offspring BW was likely related to the high rates of inactive women and few women with more than 150 min/week. The results of a meta-analysis suggested that changes in birth size outcomes are dependent on the intensity of LTPA, with larger effects observed with higher intensity. The authors raised the hypothesis that LTPA intensity needs to reach a certain threshold before some effect on nutrient supply to the fetus [16]. However, the use of a validated questionnaire for Brazilian pregnant women [31] could allow for reliable overall estimates of habitual LTPA. Second, our study population did not include women living in rural areas, and further investigation is needed in rural populations. Finally, due to our sample size, we performed mediation analyses considering LTPA in the second or third trimester of pregnancy as the exposure. Future studies should explore the mediating effect of GWG in either the second or third trimester of pregnancy.

The findings of this study support those of a recent meta-analysis [16], which showed that LTPA in late, but not in early, pregnancy reduces offspring BW. However, over 70% of the total sample size of studies included in that meta-analysis were participants of the Danish National Birth Cohort, which includes only white women, and almost 11% had gestational diabetes mellitus. Most existing evidence for the relationship between LTPA during pregnancy and offspring BW was reported by studies from high income countries. The present study was conducted in a low-income area with high proportion of socially vulnerable women. Although other studies reported similar findings as those of the present study, it is difficult to compare the results due to differences in questionnaires used to evaluate LTPA. For example, in the Norwegian Mother and Child Cohort Study (43,705 pregnancies), exercise during pregnancy was associated with a decrease in offspring BW of 2.9 grams per unit of increase in exercise (one time per month) [40]. Another cohort study, in Michigan, United States, revealed that aerobic physical activity was inversely associated with foetal growth ratio [41]. Babies born to white American women in the highest quartile of physical activity weighed 608 grams less than babies born to white women in the lowest quartile. Although accelerometers were used in that study to estimate physical activity in pregnancy, the sample size was very small (n = 51) [41]. Conversely, findings of a meta-analysis that was able to consider intensity of physical activity suggested that moderate and high levels of physical activity were directly and inversely associated with BW, respectively [42].

The association between LTPA during pregnancy and offspring BW was stronger among adult women in our study, and the association was not significant among adolescents. However, the sample size of adolescents may have been too small to explore this relationship. We also found that excessive weight gain significantly mediated the association between LTPA and BW by reducing offspring BW. However, this effect was small. In previous studies, the LTPA during pregnancy reduced the risk of excessive GWG [11, 12]; in turn, GWG above the recommendations increased the risk of inadequate birth weight [43], suggesting the excessive maternal weight as a mediator between the LTPA and BW relationship, as we found in our study population. There was no evidence suggesting any role of insufficient GWG in the association between LTPA during pregnancy and offspring BW. Similarly, a previous meta-analysis [16] reported that associations remained significant after adjustment for maternal BMI, possibly suggesting that the effect of physical activity on offspring BW is only partially mediated by maternal weight. Increasing physical activity during pregnancy promotes fetal growth [44] and increases maternal insulin sensitivity independent of maternal weight [45]. This is a result of reduced glucose availability to the foetus due to an increased uptake by active muscles [44]. We may thus infer that the recommended levels of LTPA should be encouraged during prenatal care. Other potential mediators, such as gestational diabetes mellitus [13] and hypertension disorders [14], should be investigated in future studies.

Despite a small number of SGA and LGA infants in this study, we explored the association of these outcomes with LTPA during pregnancy. No significant associations were observed, which is similar to a recent meta-analysis indicating that physical activity was not associated with SGA [16]. However, a longitudinal study of 826 American mother-neonate pairs found an increased risk of SGA with the highest quartile of physical activity compared to the lowest level [46]. In the Danish National Birth Cohort (n = 79,629), a decreased risk of SGA was found in pregnant women who exercised compared to those who were sedentary (hazard ratio, 0.87; 95% CI: 0.83,0.92) [47]. Another Brazilian cohort study did not find statistically significant associations between high or low levels of physical activity during pregnancy and low offspring BW [48].

LGA infants have higher risks of obesity and raised metabolic disease markers in childhood compared to babies with adequate BW [49, 50]. Thus, determining whether physical activity during pregnancy can reduce the risk of the infant being born LGA is important. Although this study found no such association, it is likely due to the small number of LGA infants in a context of obstetric low-risk pregnancies. A meta-analysis published in 2019 by Pastorino et al. [16] indicated that physical activity in the third trimester of pregnancy is consistently associated with a modestly lower risk of LGA.

In conclusion, this study found that a minimum of 150 min/week of LTPA in the third trimester of pregnancy, but not in the second trimester of pregnancy, was negatively associated with offspring BW in an Amazonian population. Excessive GWG, but not insufficient GWG, mediated the effect between LTPA in the third trimester of pregnancy and offspring BW, reducing BW by 34.7 grams. These results provide further evidence for the beneficial effect LTPA during pregnancy on preventing excessive offspring weight gain.

## Supporting information

S1 Table Percentage of pregnant women according to levels of physical activities in the second and third trimesters of pregnancy in the MINA-Brazil cohort. (DOCX)

S2 Table Effect of 150 min/week of moderate to intense leisure-time physical-activity (LTPA) during pregnancy on offspring birth weight in the MINA-Brazil cohort. (DOCX)

S3 Table Effect of leisure-time physical activity (LTPA) during pregnancy on offspring birth weight z-score for gestational age stratified by maternal age and gestational weight gain in the MINA-Brazil cohort study. (DOCX)

## Abbreviations

BMI: body mass index
BW: birth weight
CDE: controlled direct effect
GWG: gestational weight gain
LGA: large-for-gestational-age
LTPA: leisure-time physical activity
MINA: the Maternal and Child Health and Nutrition in Acre study
NIE: natural indirect effect
SGA: small-for-gestational-age
TE: total effect
WHO: World Health Organization
