# Supplementary material for: Leisure-time physical activity in Amazonian pregnant women and offspring birth weight: A prospective cohort study
Source: PLoS One. 2022 Mar 16;17(3):e0265164. doi: 10.1371/journal.pone.0265164 (PMC8926278; doi:10.1371/journal.pone.0265164)
Supplement: S1 Table — (DOCX) [file pone.0265164.s001.docx]

**S1 Table. Percentage of pregnant women according to levels of physical activities in 2^nd^ or 3^rd^ trimester of** **pregnancy in the MINA-Brazil cohort.**

| **Levels of physical activities** | **N** | **%** |
| --- | --- | --- |
| Inactive^a^ *during 2^nd^ trimester of pregnancy^b^* |  |  |
| Yes | 364 | 80.0 |
| No | 91 | 20.0 |
| Inactive^a^ *during 3^rd^ trimester of pregnancy^b^* |  |  |
| Yes | 324 | 71.7 |
| No | 128 | 28.3 |
| *Highly^c^ active during 2^nd^ trimester of pregnancy^b^* |  |  |
| Yes | 11 | 2.4 |
| No | 444 | 97.6 |
| *Highly^c^ active during 3^rd^ trimester of pregnancy^b^* |  |  |
| Yes | 11 | 2.4 |
| No | 441 | 97.6 |
| *Achieved* 150 min/week *of LTPA in 2^nd^ trimester of pregnancy^b^* |  |  |
| Yes | 33 | 7.3 |
| No | 422 | 92.7 |
| *Achieved* 150 min/week *of LTPA in 3^rd^ trimester of pregnancy^b^* |  |  |
| Yes | 43 | 9.5 |
| No | 409 | 90.5 |
| *Achieved* 150 min/week *of LTPA in 2^nd^ or 3^rd^ trimester of pregnancy^b^* |  |  |
| Yes | 66 | 13.2 |
| No | 434 | 86.8 |
| *Achieved 150 min/week of moderate to intense LTPA* *2^nd^ trimester of* pregnancy^b^ |  |  |
| Yes | 19 | 4.2 |
| No | 436 | 95.8 |
| *Achieved 150 min/week of moderate to intense LTPA* in *3^rd^ trimester of* pregnancy^b^ |  |  |
| Yes | 30 | 6.6 |
| No | 422 | 93.4 |
| *Achieved 150 min/week of moderate to intense LTPA* in *2^nd^ or 3^rd^ trimester of* pregnancy^b^ |  |  |
| Yes | 44 | 8.8 |
| No | 456 | 91.2 |

^a^Inactive is not getting any moderate- or vigorous-intensity physical activity beyond basic movement from daily life activities [32].

^b^*2^nd^ trimester of* pregnancy: mean 19.6 (SD 2.4) weeks of pregnancy; *3^rd^ trimester of* pregnancy: mean 27.8 (SD 1.6) weeks of pregnancy.

^c^Highly active is doing the equivalent of more than 300 minutes of moderate-intensity physical activity a week. This level exceeds the key guideline target range for adults.
