# Supplementary material for: Leisure-time physical activity in Amazonian pregnant women and offspring birth weight: A prospective cohort study
Source: PLoS One. 2022 Mar 16;17(3):e0265164. doi: 10.1371/journal.pone.0265164 (PMC8926278; doi:10.1371/journal.pone.0265164)
Supplement: S2 Table — (DOCX) [file pone.0265164.s002.docx]

**S2 Table. Effect of 150 min/week of moderate to intense leisure-time physical-activity (LTPA) during pregnancy on offspring birth weight in the MINA-Brazil cohort.**

| *Achieved 150 min/week of*  *moderate to intense LTPA* |  | **Offspring birth weight (in z-score for gestational age)^b^** | | | |
| --- | --- | --- | --- | --- | --- |
|  |  | Crude Model |  | Adjusted Model^c^ |  |
|  | N (%) | β (95% CI) |  | β (95% CI) |  |
| *2^nd^ trimester of* pregnancy^a^ |  |  |  |  |  |
| Yes | 19 (4.2) | -0.41 (-0.86; 0.04) |  | -0.28 (-0.71; 0.16) |  |
| No | 436 (95.8) | Reference |  | Reference |  |
| *3^rd^ trimester of* pregnancy^a^ |  |  |  |  |  |
| Yes | 30 (6.6) | **-0.45 (-0.81; -0.10)** |  | **-0.45 (-0.79; -0.11)** |  |
| No | 422 (93.4) | Reference |  | Reference |  |
| *2^nd^ or 3^rd^ trimester of* pregnancy^a^ |  |  |  |  |  |
| Yes | 44 (8.8) | **-0.48 (-0.78; -0.19)** |  | **-0.43 (-0.71; -0.14)** |  |
| No | 456 (91.2) | Reference |  | Reference |  |
|  |  | **Offspring birth weight (in grams)^d^** | | | |
|  |  | Crude Model |  | Adjusted Model^c^ |  |
|  | N (%) | β (95% CI) |  | β (95% CI) |  |
| *2^nd^ trimester of* pregnancy^a^ |  |  |  |  |  |
| Yes | 19 (4.2) | -161.7 (-403.9; 80.5) |  | -95.6 (-275.5; 84.3) |  |
| No | 436 (95.8) | Reference |  | Reference |  |
| *3^rd^ trimester of* pregnancy^a^ |  |  |  |  |  |
| Yes | 30 (6.6) | **-304.7 (-494.4; -115.0)** |  | **-174.0 (-317.1; -31.0)** |  |
| No | 422 (93.4) | Reference |  | Reference |  |
| *2^nd^ or 3^rd^ trimester of* pregnancy^a^ |  |  |  |  |  |
| Yes | 44 (8.8) | **-260.36 (-418.4; -102.4)** |  | **-167.4 (-285.2; -49.5)** |  |
| No | 456 (91.2) | Reference |  | Reference |  |

^a^*2^nd^ trimester of* pregnancy: mean 19.6 (SD 2.4) weeks of pregnancy; *3^rd^ trimester of* pregnancy: mean 27.8 (SD 1.6) weeks of pregnancy.

^b^Z-scores of birth weight for gestational age calculated according to the Intergrowth-21st Project standard [34].

^c^Adjusted model: adjusted by determinants in distal level (number of rooms in the household, household wealth index, living with a partner); intermediate level (pre-pregnancy body mass index, age, primigravida), and proximal level (frequency of fruit and vegetable consumption and ultra-processed food consumption, smoking during pregnancy).

^d^Controlled for newborn sex and gestational age at delivery.
