# Supplementary material for: Leisure-time physical activity in Amazonian pregnant women and offspring birth weight: A prospective cohort study
Source: PLoS One. 2022 Mar 16;17(3):e0265164. doi: 10.1371/journal.pone.0265164 (PMC8926278; doi:10.1371/journal.pone.0265164)
Supplement: S3 Table — (DOCX) [file pone.0265164.s003.docx]

**S3 Table. Effect of leisure-time physical activity (LTPA) during pregnancy on offspring birth weight z-score for gestational age stratified by maternal age and gestational weight gain in the MINA-Brazil cohort study.**

|  | Offspring birth weight z-score for gestational age^b^ | | | |
| --- | --- | --- | --- | --- |
|  | Crude model | | Adjusted model^c^ |  |
|  | Maternal age | | |  |
| Adults (≥ 19 years) | β (95% CI) |  | β (95% CI) |  |
| LTPA in *2^nd^ or 3^rd^ trimester of* pregnancy^a^ (n = 392) |  |  |  |  |
| 150 minutes per week (n = 48) | **-0.46 (-0.74; -0.17)** |  | **-0.44 (-0.71; -0.17)** |  |
| Less than 150 minutes per week | Reference |  | Reference |  |
| Adolescents (< 19 years) |  |  |  |  |
| LTPA in *2^nd^ or 3^rd^ trimester of* pregnancy^a^ (n = 108) |  |  |  |  |
| 150 minutes per week (n = 18) | -0.01 (-0.52; 0.50) |  | -0.25 (-0.81; 0.30) |  |
| Less than 150 minutes per week | Reference |  | Reference |  |
|  | Gestational weight gain | | |  |
| Adequate |  |  |  |  |
| LTPA in *2^nd^ or 3^rd^ trimester of* pregnancy^a^ (n = 136) |  |  |  |  |
| 150 minutes per week (n = 24) | **-0.43 (-0.85; -0.01)** |  | -0.41 (-0.83; 0.01) |  |
| Less than 150 minutes per week | Reference |  | Reference |  |
| Insufficient |  |  |  |  |
| LTPA in *2^nd^ or 3^rd^ trimester of* pregnancy^a^ (n = 138) |  |  |  |  |
| 150 minutes per week (n = 20) | -0.00 (-0.45; 0.45) |  | -0.01 (-0.43; 0.46) |  |
| Less than 150 minutes per week | Reference |  | Reference |  |
| Excessive |  |  |  |  |
| LTPA in *2^nd^ or 3^rd^ trimester of* pregnancy^a^ (n = 188) |  |  |  |  |
| 150 minutes per week (n = 17) | -0.45 (-0.93; 0.03) |  | **-0.48 (-0.95; -0.01)** |  |
| Less than 150 minutes per week | Reference |  | Reference |  |

^a^2^nd^ trimester of pregnancy: mean 19.6 (SD 2.4) weeks of pregnancy; 3^rd^ trimester of pregnancy: mean 27.8 (SD 1.6) weeks of pregnancy.

^b^Z-scores of birth weight for gestational age calculated according to the Intergrowth-21st Project standard [36].

^c^Adjusted model: adjusted by determinants in distal level (number of rooms in the household, household wealth index, living with a partner); intermediate level (pre-pregnancy body mass index, age, primigravida), and proximal level (frequency of fruit and vegetable consumption and ultra-processed food consumption, smoking during pregnancy).
